# A Qualitative Investigation on Chronic Disease Management and Prevention Among Older Adults During the COVID-19 Pandemic

**DOI:** 10.1177/08901171231218681

**Published:** 2023-11-24

**Authors:** Michelle C. Yang, Cam Clayton, Devin Harris, Chelsea Pelletier, Julia Schmidt, Jill G. Zwicker, Brodie M. Sakakibara

**Affiliations:** 1 Graduate Programs in Rehabilitation Sciences, University of British Columbia, Vancouver, BC, Canada; 2 Centre for Chronic Disease Prevention and Management, University of British Columbia, Kelowna, Canada; 3 Vancouver Fraser Medical Program, University of British Columbia, Vancouver, BC, Canada; 4 Department of Emergency Medicine, University of British Columbia, Vancouver, BC, Canada; 5 Interior Health Authority, Kelowna, BC, Canada; 6 School of Health Sciences, 6727 University of Northern British Columbia, Prince George, BC, Canada; 7 Department of Occupational Science and Occupational Therapy, University of British Columbia, Vancouver, BC, Canada; 8 Department of Pediatrics, University of British Columbia, Vancouver, BC, Canada; 9 BC Children’s Hospital Research Institute, Vancouver, BC, Canada

**Keywords:** chronic disease prevention and management, self-management, COVID-19, older adults, qualitative research, motivation

## Abstract

**Purpose:**

To qualitatively describe experiences of chronic disease management and prevention in older adults (age ≥65 years) during COVID-19.

**Approach:**

Qualitative descriptive approach.

**Setting:**

Data collected online via telephone and video-conferencing technologies to participants located in various cities in British Columbia, Canada. Data analyzed by researchers in the cities of Vancouver and Kelowna in British Columbia.

**Participants:**

Twenty-four community-living older adults (n = 24) age ≥65 years.

**Methods:**

Each participant was invited to complete a 30-to-45-minute virtual, semi-structured, one-on-one interview with a trained interviewer. Interview questions focused on experiences managing health prior to COVID-19 and transitioning experiences of practicing health management and prevention strategies during COVID-19. Audio recordings of interviews were transcribed verbatim and analyzed thematically.

**Results:**

The sample’s mean age was 73.4 years (58% female) with 75% reporting two or more chronic conditions (12.5% none, 12.5% one). Three themes described participants’ strategies for chronic disease management and prevention: (1) having a purpose to optimize health (i.e., managing health challenges and maintaining independence); (2) internal self-control strategies (i.e., self-accountability and adaptability); and (3) external support strategies (i.e., informational support, motivational support, and emotional support).

**Conclusion:**

Helping older adults identify purposes for their own health management, developing internal control strategies, and optimizing social support opportunities may be important person-centred strategies for chronic disease management and prevention during unprecedented times like COVID-19.

## Purpose

The aging population is at increased risk of developing chronic diseases (e.g., stroke, cardiovascular diseases, diabetes, etc.).^1-3^ In Canada, 73% of individuals aged 65 years and older are reported to have at least one chronic disease.^4^ Evidence also indicates that 33% of the Canadian population have multimorbidity.^5^ The health needs of people are changing, and there is an increase in demand to support older individuals in managing their chronic diseases to improve their functioning, quality of care, and overall well-being.^6,7^

Chronic disease management and prevention was especially important during the COVID-19 pandemic, as public health prevention strategies to reduce viral spread (i.e., quarantine, social and physical distancing, and closure of public facilities) increased barriers to managing health and wellness.^8^ For example, social isolation and physical distancing are associated with poor mental health and increased stress, which in turn increase the risk of heart diseases.^9^ Moreover, closures of public areas (e.g., parks, shopping centres) act as barriers to exercise and dietary quality by limiting the options of where to obtain physical activity (i.e., facility access) and when people can leave the house.^10,11^ While COVID-19 prevention strategies are necessary, they could increase the risk of adverse health events and have negative effects on the management of health-related behaviours, which are established risk factors for many chronic diseases. For example, those with pre-existing medical conditions (e.g., chronic diseases) are at higher risk of contracting the virus and developing more severe outcomes.^12^ During the pandemic, older adults experienced decreases in physical activity, increases in anxiety and depression, and poorer sleep quality.^8,10,11^ While there is literature on chronic disease management and prevention during COVID-19, it is primarily focused on the provision of management and prevention supports using telehealth strategies.^13–15^ There is a lack of research on the lived-experiences of how people managed their health and prevented disease and disability during times of social and physical distancing, such as during the COVID-19 pandemic. Thus, the objective of this paper is to qualitatively describe the chronic disease management and prevention experiences of older adults (≥65 years of age) during the initial phases of the COVID-19 pandemic.

## Methods

### Approach

A qualitative description paradigm^16,17^ was used to develop an understanding of chronic disease management and prevention among adults (≥65 years of age) during COVID-19. The COREQ (COnsolidated criteria for REporting Qualitative research) checklist was used to ensure comprehensive reporting of our qualitative methodologies and findings (Supplementary Material 1).^18^ Ethical approval was obtained from the Behavioural Research Ethics Board (H20-01368) at the University of British Columbia (UBC).

### Setting and Participants

In this qualitative evaluation of data from a larger health promotion study of older adults during COVID-19, community-dwelling individuals from British Columbia (BC), Canada were included in the study if they: (1) were 65 years or older; (2) had access to a telephone or video-conferencing program; and (3) had no previous COVID-19 diagnosis by health professionals. Individuals were excluded if they: (1) were not medically stable (e.g., symptoms, conditions, illnesses that are not being treated); (2) were participating in another health promotion program; and/or (3) had severe hearing loss that could not be corrected with a hearing aid.^19^ Participants were recruited between November 2020 to April 2021, provided virtual eConsent through an online Qualtrics consent form, and participated in interviews using Zoom videoconferencing. Based on previous literature reporting on differences in health management by sex and geographic location, we used stratified purposive sampling to ensure representation of both male and female sexes, and different geographical locations of residence (e.g., urban, rural/suburban).^20-23^ Potential study participants were invited to participate in this qualitative study at the end of their participation in a previous study. Study participants were recruited until the research team determined that data sufficiency had been reached (i.e., redundancy in observations).^24,25^ As previously recommended in the literature, we estimate that a sample size of 20 participants from the program will suffice.^26^ Twenty participants were initially recruited and interviewed, and then interviews went through initial analysis steps. A few more participants were then recruited one by one and coded after each interview to ensure that no outstanding findings were identified. In total, 24 participants were included in this study to reach data sufficiency, with no dropouts or refusals.

### Data Collection

Participants were interviewed using an open-ended semi-structured interview guide developed by the research team. It consisted of questions related to experiences in managing health and preventing health challenges prior to COVID-19, and transitioning experiences of health management and prevention during COVID-19 (Supplementary Material 2). The interview guide was further refined to include any new topics that emerge throughout interviews. The virtual interviews took approximately 30 to 45 minutes to complete and were audio-recorded. Participants were interviewed on a one-to-one basis by the primary author, who had no personal relationships with any of the participants. The interviewer was trained by the principal investigator to deliver and lead the open-ended interview sessions. The interviewer was a 24-year-old female rehabilitation sciences graduate student with an interest in chronic disease management and prevention, health promotion, and telehealth. As a graduate student, she had the opportunity of receiving the training and education to prepare for her role as an interviewer and researcher. Her experience researching this field for her graduate studies may be a source of bias, therefore probing questions were asked for clarification while speaking to interviewees to limit both subjectivity and bias. Supplementary field notes were also written throughout the interview process.

### Analysis

A data-driven codebook thematic analysis was conducted for this study, which adapts an iterative process outlined while also using a structured coding approach to support the analysis process.^27,28^ Analysis began with the primary author and a research assistant transcribing the data verbatim and reading the interview transcripts. The research assistant was an undergraduate student with a background in psychology and an interest in chronic care, telehealth, and self-management. Documentation of theoretical and reflective thoughts was done for data immersion.^29^ The primary author and research assistant then identified, categorized, and sorted important sections of text into codes using NVivo-12.^27^ Coding began with each team member coding the same first few transcripts independently and then discussed findings together to establish any similar thoughts and findings.^30^ A coding guide was then developed to organize and manage each code. Codes were not drawn from pre-existing ideas developed prior to the analysis process. The guide was modified throughout the coding process when additional key elements were identified in the data. The remaining transcripts were then divided and coded separately by the two team members. Regular meetings were held to continue discussion of findings throughout the analysis process. Next, codes were clustered into categories, which were then sorted into themes through a collaborative process with the primary author, the principal investigator, and the same research assistant. A theme was identified if it brought “meaning and identity to a recurrent experience and its variant manifestations” and captured something important about the objective.^31,32^ By the end of analysis, themes relevant to the research objectives were created, named, and defined.

Participants were emailed a one-page summary of the findings and request to comment or clarify any points. All participants confirmed that the summary accurately reflected their thoughts. Reflexivity was noted during each analysis process (i.e., interviewing, transcribing coding, theme development) to monitor the interviewer’s positions, values, beliefs, or biases that may have influenced data interpretation.^33,34^ All records of raw data, field notes, and transcripts were kept for researchers to clearly systemize, relate, and cross-reference data.^35^

## Results

Ten males and fourteen females participated in this study. The mean age was 73.1 years (SD 7.4) for male participants and 74.1 years (SD 5.6) for female participants. Among male participants, 29% percent reported having more than one chronic condition and 25% lived in urban regions. Among female participants, 46% reported having more than one chronic condition, 8% reported having no chronic conditions, and 21% lived in an urban area. Table 1 further details the sample characteristics. Based on our interpretation of the interviews, we did not find any salient differences in chronic disease management and prevention between respondents of different sex or from different geographic regions. No participants were diagnosed with COVID-19 during participation in the larger health promotion study.

**Table 1.** Sample Characteristics by Sex (n = 24).

Sample Characteristics	n (%) or Mean ± SD	
	Male (n = 10)	Female (n = 14)
Age (years)	73.1±7.4	74.1±5.6
Geographical Region (within British Columbia, Canada)		
Urban regions	6 (25)	5 (21)
Rural/suburban regions	4 (16)	9 (38)
Chronic Diseases		
0	1 (5)	2 (8)
1	2 (8)	1 (5)
More than 1	7 (28)	11 (46)
Race/Ethnicity		
White or Caucasian	9 (38)	11 (46)
Other	1 (4)	3 (13)
Marital Status		
Married/common law	7 (29)	6 (25)
Other	3 (13)	8 (33)
Family Size		
1 (Living Alone)	3 (13)	8 (33)
2 or more	7 (29)	6 (25)
Employment Status		
Employed (working full-time/part-time)	2 (8)	1 (4)
Retired	8 (33)	12 (50)
Other	0	1 (4)
Highest Education Level/Degree		
High school degree or equivalent (i.e., GED)	1 (4)	1 (4)
Bachelor’s degree (i.e., BA, BSc)	3 (13)	4 (17)
Other (i.e., Graduate, technical degrees, etc.)	6 (25)	9 (38)
Approximate Gross Household Income		
Less than $50,000	6 (25)	7 (29)
$50,000 - $79,999	0	2 (8)
$80,000 or more	3 (13)	3 (13)
Prefer not to answer	1 (4)	2 (8)

Our findings revolve around management and prevention strategies through modification of health behaviours (e.g., physical activity, diet, mental health state, social interaction). Three overarching themes were identified that align with participants’ experiences in using different management and prevention strategies to modify health behaviours during COVID-19: (1) having a purpose to optimize health (e.g., managing health challenges, maintaining independence); (2) using internal self-control strategies (e.g, self-accountability, resilience); and (3) relying on external support strategies (e.g., informational, motivational, emotional support).

### Theme 1: Having a Purpose to Optimize Health

Participants described the importance of having a purpose to optimize their health for chronic disease management and prevention. Individuals finding their own purpose to practice their specific health behaviours allowed them to gain the encouragement needed for both health management and disease prevention. How participants gained this encouragement tended to be situational, with participants finding their own reasons through specific events (e.g., chronic disease onset) in their lives. Two subthemes were identified to explain participants’ purposes: “managing health challenges” and “maintaining independence”.

#### Managing Health Challenges

Participants expressed wanting to practice better health behaviours to either ensure no future occurrences of secondary issues or prevent any new health challenges. Some participants dealt with underlying health challenges prior to participating in the study (e.g., high blood pressure, diabetes, arthritis) and focused more on management, as represented by a 77-year-old male in an urban region: *“…it’s been important to me for quite a while to try and [manage my health] … since I was diagnosed with coronary artery disease… I began to take it a bit more seriously.”* Other participants did not have any underlying health issues coming into the study, and thus focused more on primary prevention (i.e., preventing health conditions before they occur). For instance, a 75-year-old male from an urban region reflected on the following: *“…I’ve seen people before…a few old guys who were probably 60…who’d been told they had to run or do exercise because they had a heart problem… I don’t want to be like that.”* Once COVID-19 emerged, participants recognized the importance of managing their health to reduce vulnerability to COVID-19. This was highlighted by an 82-year-old male in an urban region that previously had a heart attack, where he mentioned, *“…even if one didn't contract COVID-19, knowing that if by chance someone did, you want to be in the best health situation you could be in, just in case….”* Whether it was to manage other health challenges or to minimize their risk of contracting COVID-19, participants identified the importance of managing their health behaviours overall.

#### Maintaining Independence

Overall, participants were aware of the importance of practicing positive health-related behaviours for chronic disease management and prevention. Many wanted to practice these behaviours because they viewed good health-related behaviours as an important contributor to maintaining independence at older ages. This was especially important for participants who lived alone, as highlighted by an 81-year-old female living in a rural/suburban region: *“My husband passed away… and I wanted to stay living by myself. So, I tried to do as much as I can myself…”* Additionally, practicing positive health behaviours would allow some participants to continue doing activities of interest, including different physical activities (e.g., running, yoga), as noted by a 74-year-old female living in a rural/suburban region: *“I’ve always been an active person. So, [physical] activities have always been part of my life from an early age.”* While COVID-19 restrictions posed limitations in activities that could be done, many participants still wanted to continue managing their health and well-being. People value the ability to maintain independence, and thus served as a purpose to optimize their health to support the continuation of their activities of interest and overall well-being, especially during COVID-19.

### Theme 2: Using Internal Self-Control Strategies

This theme focused on innate strategies that participants used for their chronic disease management and prevention. Two subthemes were identified that represented participants’ internal self-control strategies: “self-accountability” and “adaptability”.

#### Self-Accountability

Self-accountability is defined as one taking responsibility for one’s actions and life^36^ and was discussed as an important strategy used to facilitate health behaviours as a means of chronic disease management and prevention. Participants felt self-accountability was especially important during COVID-19 because quarantining, social isolation and physical distancing strategies caused many to stay at home by themselves for longer periods of time. Some participants discussed continuing to hold themselves accountable when, for instance, having a poor diet or decreased physical activity during the winter. Participants would keep themselves accountable by assuring that they return to better health behaviours the following seasons. Similarly, participants reported that at times they would have unhealthy behaviours (e.g., poor diet, lack of physical activity) due to staying at home more often during COVID-19, but they made sure to eventually resume their healthy behaviours via constant self-reminders, as highlighted by a 65-year-old male on living in a rural/suburban region: *“You have to keep [healthy behaviours] you just can't go for two or three months doing, you know, doing your exercises and eating healthy all the time…”.* On the other hand, while participants noted the importance of keeping themselves accountable, some participants mentioned struggling to keep that accountability during COVID-19. Specifically, one 73-year-old female from an urban region reflected on her struggles in motivating herself:

I tried to find alternative exercises, or activities, but they’re not comparable to what I was doing before COVID…exercising at home…I probably exercise like thirty minutes a day but I was doing four hours a week before so that’s not enough…it’s so hard to motivate yourself when you’re doing it by yourself.

Overall, whether participants were able to continue practicing this strategy successfully or not during COVID-19, self-accountability was an overarching strategy that participants attempted to implement to practice their health behaviours at home.

#### Adaptability

Participants explained how they adapted to different challenges during COVID-19 that interfered with their ability to engage in positive health behaviours. For participants, these challenges came in the form of health difficulties, such as depression and anxiety, and conflicting life roles, such as occupational duties. Due to these challenges, participants discussed the need to be adaptive, whereby they would readjust their schedules to incorporate physical activity or postpone the activities to another day. For example, a 69-year-old female from a rural/suburban region had ongoing mental health challenges interfering with her other health-related activities: *“Well, if it’s my mental health, I isolate myself [and don’t go on my daily walks] … hoping that the next day would be better if I had a bad day [and then I can continue my walk the next day].”* Participants who still had ongoing occupations (e.g., full-time job) discussed challenges finding time to manage their health behaviours within their busy schedules. For example, one 68-year-old female from a rural/suburban region had difficulties finding time to incorporate healthy activities because of her full-time job: *“[I could] go on days without really thinking about my health and what my needs might be… Running on the treadmill of life…”*

While participants constantly attempted to keep themselves accountable and maintain the health behaviour strategies they implemented into their lives, some expressed difficulties re-incorporating positive health behaviours because COVID-19 limited the type of health activities in which they could participate. Specifically, participants who relied on activities offered at community centres, such as yoga and weightlifting, felt limited in activities during COVID-19. For instance, a 77-year-old female from a rural/suburban region stated, *“…I have never done much yoga at home. I could… But I’ve never done it very much at home…”* Another participant, a 65-year-old male from an urban region, attempted to adapt to the COVID-19 restrictions by replacing his swimming activities with walking: *“The restriction…swimming is a big thing for me and you cannot go to community center… but I tried to overturn them on the walking.”* These restrictions introduced a challenge for participants’ self-accountability, as limitation of activities leaves them with little viable options for practicing health activities that they want to do. Thus, despite these situational changes, participants still attempted to keep themselves accountable and maintain positive health behaviours during COVID-19 when possible.

### Theme 3: Relying on External Support Strategies

Participants identified social support as an important means to facilitate chronic disease management and prevention. It was especially important during COVID-19, when ongoing restrictions limited options for people to gain social support. Participants attempted to seek external supports during the pandemic while adhering to public restrictions due to COVID-19. Specifically, participants highlighted the importance of (subthemes): informational, motivational, and emotional support.

#### Informational Support

Informational support that participants sought included information on current health challenges and additional resources to maintain their health management. This information was obtained from external sources, such as health professionals (e.g., family physicians). Some participants interacted regularly with their family physicians to obtain information about their current health conditions. For instance, a 77-year-old male from a rural/suburban region mentioned, *“…there were things that I would see my doctor about [regarding my diet or other areas of my health] and she would recommend around any particular [health-related] area that I’m interested in [understanding how to improve].”* Furthermore, participants reported obtaining informational support from members of their community (e.g., community centre members), which included suggestions for various activities in which they could engage that would be beneficial for their health management. Particularly, a 73-year-old female from an urban region would attend physical activity classes in her community centre and obtain informational resources from community members: *“…a friend I met at the [exercise] classes suggested that I take another [type of exercise] class [that would benefit my physical activity goals] …”* Thus, through attending physical activity classes at her community centre, she was able to gain information on other physical activity classes to further support her physical health journey. The utilization of informational supports was especially important when COVID-19 occurred; participants reported on how receiving information (e.g., regarding classes, programs, and other resources to help them with health-related areas they were interested in managing) from others was beneficial especially during COVID-19 when safety guidelines limited the options and availability for health-related activities. For example, one 85-year-old female from a rural/suburban region was recommended an online exercise class that she used to help her continue regular physical activity during the pandemic: *“A friend recommended one site and it wasn’t bad…I [would] do it every two or three days…”* Overall, obtaining informational support from external sources allowed participants to learn about new resources to support their health behaviours for chronic disease management and prevention, especially during COVID-19.

#### Motivational Support

Participants reported seeking motivational support from close social connections to both initiate and sustain chronic disease management and prevention practices. Some participants highlighted how close friends or family that lived alongside them acted as models to become motivated to continue with healthy behaviours during COVID-19. A 68-year-old female participant from a rural/suburban region often used her physically active daughter as an example to motivate herself to be just as physically active: *“… my daughter’s very fit and leads a very healthy lifestyle…she was definitely a big motivator for me to remind me to pay more attention to a healthier lifestyle.”* During COVID-19, a 76-year-old female in an urban region also mentioned meeting with two people from her running group as part of one of their regular weekly physical activities: *“…we decided let’s go get back running again, but it’ll just be the three of us and we can be at a distance….”* The smaller group size allowed them to continue their running activities while practicing physical distancing, and thus allowed them to provide each other motivational support during the pandemic. However, not all participants found it easy to keep those close social connections. A 77-year-old female from a rural/suburban region mentioned how not having a community to do activities with in-person created a challenge for her as someone who lived alone:

It was a great challenge, and I really found that not having those activities, which also got me out of the house and which connected me with a community of people…in yoga a community of people that I’ve known really well for a long time…it was very upsetting in the beginning…

While some had close friends and family that were in their respected social bubble to provide motivation, others had a challenge gaining that external motivation due to COVID-19 restrictions limiting the ways they could gain those social connections.

#### Emotional Support

Family and friends also offered emotional support to participants when dealing with stresses and other issues. One 76-year-old female participant from an urban region highlighted how her family’s support helps her to continue managing her health: *“I think it’s a little bit difficult [managing my health],but I do have a good support for my family. It makes a difference. I try to keep my mental me happy and positive…people need people.”* At the beginning of COVID-19, some participants noted the value of having close friends or family members living nearby to help manage their mental health. A 65-year-old female and a 66-year-old male from rural/suburban regions both mentioned the value of having family members that lived with them to support their mental well-being: *“…we were living in the same house with my daughter and her husband, so we have a little bubble…”* Having two other family members in the household with them provided enough positive social interaction for both participants to feel emotionally supported during COVID-19, especially for their mental health.

## Discussion

In this study, we aimed to develop a greater understanding of chronic disease management and prevention experiences of older adults during COVID-19. Our findings highlight important elements that occur in individuals’ own journeys of chronic disease management and prevention. At the foundation of chronic disease management and prevention, our findings suggest that people need a purpose to manage their health conditions or prevent new health challenges from occurring (“having a purpose to optimize health”). Once a purpose has been identified, participants rely on internal senses of control (“internal self-control strategies”), followed by engaging in external support resources (“external support strategies”). These were all used during COVID-19, but with some adapted actions due to limitations in accessing public spaces and services (e.g., practicing physical activity at home) during lockdown periods. People also needed to be more intentional in seeking social support, as COVID-19 restrictions also limited options to obtain social interactions.

Our findings for chronic disease management align with previous studies that have reported on different processes involved in self-management.^37-41^ Particularly, our findings align with Schulman-Green et al.’s (2012) identification of three self-management processes in a systematic review of 101 studies.^41^ Two processes, “focusing on illness needs” (i.e., learning about chronic conditions, taking ownership of health needs, and performing health promotion activities) and “living with a chronic illness” (i.e., coping with a chronic condition and integrating illness needs into the context of the individual’s life), mirror our findings of an individual needing a purpose to prevent and manage chronic diseases and utilizing internal resources to manage their own health.^41^ Recognizing the importance of social support as an external strategy also fits with the third process, “activating resources” (i.e., using individuals and community resources/services that help individuals achieve their most optimal self-management ability).^41^

“Having a purpose to optimize health” can be identified and represented through a variety of actions when individuals’ initially realize why they want to being thinking about their health. This was represented in our findings by participants expressing their desire to either manage their current health challenges or maintain their independence. Previous literature has also reflected these similar points, demonstrating how individuals provide their own reasons to manage health or practice prevention through learning about their chronic illnesses and learning how to take ownership of their health needs.^41,42^ These align with our findings, as it explains that individuals recognize areas of their health that need improvement, especially if they have any health challenges that are currently interfering with their daily lives. This mindset was also reported during COVID-19, as participants continued to find a purpose to manage their health and find ways to adapt to changes in their daily lives so that they could both continue to manage their health and follow COVID-19 restrictions. In addition, previous literature on self-management also considers actions of adjusting to any health-related life changes, or reframing of expectations, priorities, and values to create meaning towards managing their health.^42,43^ This is consistent with our findings, as these actions can contribute to developing individuals’ purpose in optimizing their health, whether it is for managing their health challenges or maintaining independence. Actions are taken to prioritize what is important to an individual, and thus supporting the realization of wanting to manage and optimize their health. With the addition of COVID-19, participants continued to look at their health priorities and realize the important of managing and optimizing their health, especially during an ongoing pandemic.

After individuals realize a need to manage and optimize their health, our findings explain how strategies are developed to manage their own health (i.e., “internal self-control strategies”, such as self-accountability and adaptability). These actions depict how individuals take ownership of their health needs, perform their health promotion activities, and modify their lifestyles to adapt to changes in their health.^41,43^ Some actions include learning to use self-management skills (e.g., goal setting, decision making, problem solving) and creating a consistent health routine while also being flexible.^41,43^ Generally, individuals vary in their willingness and ability, as well as how they decide to manage their health or practice prevention.^37-45^ Individuals attempt to modify their daily lives by balancing any meaningful activities they may have while paying appropriate attention to any health-related needs.^45,46^ Strategies that individuals choose to use can also vary over time as they develop their own personal resources and skills and change any subsequent health-related needs in their lives.^45,46^ Overall, the use of internal self-control strategies summarizes the various strategies and personal skills individuals implement into their daily lives to support their self-management journey.

Finally, individuals reported engaging various types of social supports, to facilitate their health management. In the literature, social support is well studied as it relates to chronic disease management and prevention.^47-49^ Previous research outlines associations between strong social support networks and increased health and wellness, as well as associations between lower levels of social activity and lower perceived health among older adults with chronic disease.^47-49^ Specifically, our findings align with Westaway’s (2005) proposed dimensions of socio-emotional social support, including emotional and affective support (i.e., availability of people and expressions of love and affection), as well as positive social interactions.^50^ In our study, such dimensions were observed when participants highlighted motivational and emotional support they receive from family, friends, and community members during COVID-19. Particularly, this was observed when people discussed being physically active alongside friends or family (e.g., running groups) or obtaining positive social interaction from family to support the management of their mental health. In both situations, social support allowed individuals to continue practicing management and prevention strategies, representing how close and caring relationships are essential for the health, well-being, and management of individuals with chronic diseases, especially during COVID-19. Our findings are also consistent with elements of informational support highlighted in Hossain’s (2021) scoping review, which defines informational support as providing advice, suggestions, and alternative actions, and summarize how informational support aids individuals’ development of new skills.^51^ This is consistent with our findings of informational support that highlight on how interactions with health professionals and other community members could increase individuals’ knowledge of their health status and skills to further their chronic disease management and prevention efforts. Overall, our findings highlight how social support may be used to create supportive relationships that can help with chronic disease management and prevention during unpredictable times like COVID-19.

Interestingly, while some differences were outlined, our findings highlight similarities in how participants go about practicing management and prevention before and during COVID-19. Early literature on COVID-19 effects on chronic disease management and prevention indicate COVID-19 having a negative impact on these practices, with individuals experiencing decreases in physical activity, changes in eating habits, and increased substance use.^52^ Participants in our findings described having similar challenges at the start of COVID-19 due to the need to follow COVID-19 restrictions. However, the methods used for chronic disease management and prevention practices do not differ that greatly in comparison to how these practices are outlines in the literature for times before COVID-19.^53^ It is possible that, while COVID-19 added extra challenges and limitations for chronic disease management and prevention practices, individuals may still be able to find alternative ways to use the same strategies they used before COVID-19 occurred. Thus, while the overall management and prevention practices seem like what individuals may have done before COVID-19, the challenges and how individuals attempted to overcome these challenges to still do these practices is what differed during COVID-19.

Telehealth added that extra support even if some individuals already had their own adjusted strategies, a solution that occurred during COVID-19.

## Limitations

There are several limitations to this study. First, social desirability bias is possible due to participants’ knowing their responses were being audio recorded and the interviewer’s presence. Second, while sufficiency was achieved for the purposes of the study evaluation, we cannot guarantee that these experiences reflect the entire older adult population, especially those from other geographical contexts. Thus, this may limit the transferability of findings. Finally, findings suggest that the sample group may have mostly focused on sharing good health-related behaviours. Participants in this qualitative study may have been more likely to be motivated to work on their healthy behaviours by volunteering for the larger health promotion study. Thus, responses regarding how participants continued to practice chronic disease management and prevention strategies could have been more positive and seem less different overall compared to pre-COVID-19. While some struggles were voiced, the voices of those had major struggles or continue to struggle with chronic disease management and prevention strategies may not have been captured in this study.

## Conclusion

Our findings suggest a sequential nature of chronic disease management and prevention of older adults during COVID-19. Programs that provide chronic disease management and prevention support to older adults during unprecedented times like COVID-19 should be person-centred, and help individuals identify purposes for their own health management, develop internal control strategies, and optimize social support opportunities.

**So What? (Implications for Health Promotion Practitioners and Researchers)**

**What is Already Known on This Topic?**

With older adults (≥65 years of age) being at an increased risk of developing at least one chronic disease, there is an importance understand chronic disease management and prevention practices in older adults. Little research has been done about chronic disease management and prevention practices for older adults during times like COVID-19.

**What Does This Article Add?**

This study highlights chronic disease management and prevention experiences in older adults during COVID-19.

**What are the Implications for Health Promotion Practice or Research?**

Findings suggest that chronic disease management and prevention support programs to older adults during unprecedented times like COVID-19 should be person-centred, and help individuals identify purposes for their own health management, develop internal control strategies, and optimize social support opportunities.

## Supplemental Material

Supplemental Material - A Qualitative Investigation on Chronic Disease Management and Prevention Among Older Adults During the COVID-19 Pandemic Supplemental Material for A Qualitative Investigation on Chronic Disease Management and Prevention Among Older Adults During the COVID-19 Pandemic by Michelle C. Yang, Cam Clayton, Devin Harris, Chelsea Pelletier, Julia Schmidt, Jill G. Zwicker and Brodie M. Sakakibara in American Journal of Health Promotion.
