# Supplementary material for: A Qualitative Investigation on Chronic Disease Management and Prevention Among Older Adults During the COVID-19 Pandemic
Source: Am J Health Promot. 2023 Nov 24;38(3):384–93. doi: 10.1177/08901171231218681 (PMC10903114; doi:10.1177/08901171231218681)
Supplement: Supplemental Material - A Qualitative Investigation on Chronic Disease Management and Prevention Among Older Adults During the COVID-19 Pandemic [file sj-pdf-1-ahp-10.1177_08901171231218681.pdf]

Supplementary Material 1. COnsolidated criteria for REporting Qualitative research (COREQ) Checklist

COREQ (COnsolidated criteria for REporting Qualitative research) Checklist

A checklist of items that should be included in reports of qualitative research. You must report the page number in your manuscript where you consider each of the items listed in this checklist. If you have not included this information, either revise your manuscript accordingly before submitting or note N/A.

Topic	Item No.	Guide Questions/Description	Reported on Page No.
**Domain 1: Research team and reflexivity**			
Personal characteristics			
Interviewer/facilitator	1	Which author/s conducted the interview or focus group?	5-6
Credentials	2	What were the researcher's credentials? E.g. PhD, MD	5-6
Occupation	3	What was their occupation at the time of the study?	5-6
Gender	4	Was the researcher male or female?	5-6
Experience and training	5	What experience or training did the researcher have?	5-6
Relationship with participants			
Relationship established	6	Was a relationship established prior to study commencement?	5-6
Participant knowledge of the interviewer	7	What did the participants know about the researcher? e.g. personal goals, reasons for doing the research	5-6
Interviewer characteristics	8	What characteristics were reported about the inter viewer/facilitator? e.g. Bias, assumptions, reasons and interests in the research topic	5-6
**Domain 2: Study design**			
Theoretical framework			
Methodological orientation and Theory	9	What methodological orientation was stated to underpin the study? e.g. grounded theory, discourse analysis, ethnography, phenomenology, content analysis	4
Participant selection			
Sampling	10	How were participants selected? e.g. purposive, convenience, consecutive, snowball	4-5
Method of approach	11	How were participants approached? e.g. face-to-face, telephone, mail, email	4-5
Sample size	12	How many participants were in the study?	4-5
Non-participation	13	How many people refused to participate or dropped out? Reasons?	4-5
Setting			
Setting of data collection	14	Where was the data collected? e.g. home, clinic, workplace	4-5
Presence of non-participants	15	Was anyone else present besides the participants and researchers?	4-5
Description of sample	16	What are the important characteristics of the sample? e.g. demographic data, date	7, Table 1
Data collection			
Interview guide	17	Were questions, prompts, guides provided by the authors? Was it pilot tested?	5-6
Repeat interviews	18	Were repeat inter views carried out? If yes, how many?	5-6
Audio/visual recording	19	Did the research use audio or visual recording to collect the data?	5-6
Field notes	20	Were field notes made during and/or after the inter view or focus group?	5-6
Duration	21	What was the duration of the inter views or focus group?	5-6
Data saturation	22	Was data saturation discussed?	5-6
Transcripts returned	23	Were transcripts returned to participants for comment and/or	7

Topic	Item No.	Guide Questions/Description	Reported on Page No.
		correction?	
**Domain 3: analysis and findings**			
Data analysis			
Number of data coders	24	How many data coders coded the data?	6-7
Description of the coding tree	25	Did authors provide a description of the coding tree?	6-7
Derivation of themes	26	Were themes identified in advance or derived from the data?	6-7
Software	27	What software, if applicable, was used to manage the data?	6-7
Participant checking	28	Did participants provide feedback on the findings?	7
Reporting			
Quotations presented	29	Were participant quotations presented to illustrate the themes/findings? Was each quotation identified? e.g. participant number	8-15
Data and findings consistent	30	Was there consistency between the data presented and the findings?	8-20
Clarity of major themes	31	Were major themes clearly presented in the findings?	8-15
Clarity of minor themes	32	Is there a description of diverse cases or discussion of minor themes?	8-15

Developed from: Tong A, Sainsbury P, Craig J. Consolidated criteria for reporting qualitative research (COREQ): a 32-item checklist for interviews and focus groups. *International Journal for Quality in Health Care*. 2007. Volume 19, Number 6: pp. 349 – 357

Once you have completed this checklist, please save a copy and upload it as part of your submission. DO NOT include this checklist as part of the main manuscript document. It must be uploaded as a separate file.

Supplementary Material 2. Qualitative Interview Guide

A. *Experiences in managing health prior to participating in the COACH program [Prior to COVID]*

To begin, I would like to ask a few questions about your experiences in managing your own health during your daily life before COVID began and before participating in the COACH program.

1. Please tell me about your experiences in trying to keep a healthy lifestyle.
   Prompts:
   a. What did you do each day to lead a healthy lifestyle? [medications, food/nutrition, exercise/physical activity, etc.]
   b. What strategies, if any, did you have to support you in leading a healthy lifestyle?

2. What challenges, if any, did you experience when you were attempting to keep a healthy lifestyle?
   Prompts:
   a. Personal challenges (i.e., mood, emotions)?
   b. Environmental challenges (i.e., community situation)?
   c. Occupational/activity challenges (i.e., roles, responsibilities, etc.)?
   d. Did you have strategies to help overcome those challenges?

3. Did you have any community resources/services that you accessed to help manage those challenges before COVID?
   Prompts:
   a. How did you find out about those resources/services?
   b. How often did you use the resource/service?
   c. How, if at all, did those community resources/services help? [knowledge, skills, confidence]

4. Is there anything else you would like to say about your experiences before COVID in terms of managing your health?

B. Transitioning experiences of health management during COVID-19

The next few questions are related to your experiences in managing your health during the COVID-19 pandemic.

1. How did the restrictions implemented to combat COVID-19 affect your health and overall lifestyle? How did the social distancing, quarantining, etc. impact you?
   Prompts:
   a. How did your health behaviours during your daily life change because of COVID-19?
   b. New challenges that may have emerged from the emergence of COVID-19? [participation in life roles, emotional challenges, etc.]
   c. How did your daily life activities change?
   d. How did your psychological health change?

2. Prior to enrolling in this program, how did you adjust to the current practices of quarantine, self-isolation, and social distancing?
